# The impact of the intestinal microbiome on human immune development and atopic disease

**DOI:** 10.1186/1710-1492-10-S1-A63

**Published:** 2014-03-03

**Authors:** Leah T Thomas, Marie-Claire Arrieta, Pedro A Dimitriu, Lisa Thorson, William W Mohn, B Brett Finlay, Stuart E Turvey

**Affiliations:** 1 Department of Microbiology & Immunology and Pediatrics, University of British Columbia, Vancouver, BC V6T 1Z4, Canada

## Background

Asthma is a chronic inflammatory disease characterized by bronchial hyper-responsiveness [1]. As the most endemic of all childhood diseases, asthma accounts for the majority of hospitalizations and school absences in children [2]. Exciting new research focuses on the involvement of the gut microbiome in asthma development. Murine studies support the hypothesis that the administration of probiotics or antibiotics during post-natal life alters the gut microbiome and ultimately the asthmatic symptoms of these mice [3-5]. This study will translate these findings into humans using stool samples obtained from the Canadian Healthy Infant Longitudinal Development (CHILD) study.

## Hypothesis

The composition of the human gut microbiome in early life influences immune system development specifically related to asthma susceptibility, and specific microbial populations protect against or promote asthma development.

## Methods

1262 children enrolled in the CHILD study with valid skin prick test and wheeze data (determined by questionnaire/clinical assessment) were grouped into four clinically relevant phenotypes: atopic wheeze, atopic non-wheeze, non-atopic wheeze, and non-atopic non-wheeze. Individuals who test positive for both wheeze and atopy are known to be at the highest risk for developing asthma versus those of the other three phenotypes [6,7]. 16S rDNA extracted and amplified from 3-month and 1-year stool samples of children in these four phenotypes was subjected to high throughput Illumina sequencing to identify common/predominant microbial populations among these children during the first year of life. Principle coordinate analysis (PCoA) will be applied to compare the microbial taxa among the four phenotypes and assess the effect of confounding factors.

## Results

PCoA will be used to compare the microbial populations among these four phenotypes. This analysis will be extended to examine the effects of confounding factors such as, mode of delivery, antibiotic exposure, feeding methods (breast milk vs. formula/solid food diet), and furred pet exposure on the gut microfloral diversity and composition relative to asthma development. These data will ultimately be compared alongside murine model data already established in the Finlay lab as well as with additional clinical data from the CHILD study regarding asthma/allergy development in these subjects at 3- and 5-years of age.

## Conclusions

This study could potentially identify the gut microbiome as a therapeutic target to prevent the development of asthma in children, perhaps through the addition of specific probiotic regimens during the first year of life [4].
